# Studies on the Mechanisms of Anti-Inflammatory Activity of Heparin- and Hyaluronan-Containing Multilayer Coatings—Targeting NF-κB Signalling Pathway

**DOI:** 10.3390/ijms21103724

**Published:** 2020-05-25

**Authors:** Hala Alkhoury, Adrian Hautmann, Bodo Fuhrmann, Frank Syrowatka, Frank Erdmann, Guoying Zhou, Sanja Stojanović, Stevo Najman, Thomas Groth

**Affiliations:** 1 Department Biomedical Materials, Institute of Pharmacy, Martin Luther University Halle Wittenberg, Heinrich Damerow Strasse 4, 06120 Halle (Saale), Germany; hala.al-khoury@student.uni-halle.de (H.A.); adrian.hautmann@pharmazie.uni-halle.de (A.H.); guoying.zhou@pharmazie.uni-halle.de (G.Z.); 2 Interdisciplinary Center of Materials Science, Martin Luther University Halle-Wittenberg, 06120 Halle (Saale), Germany; bodo.fuhrmann@cmat.uni-halle.de (B.F.); frank.syrowatka@cmat.uni-halle.de (F.S.); 3 Pharmaceutical Biology and Pharmacology Department, Institute of Pharmacy, Martin Luther University Halle Wittenberg, Wolfgang-Langenbeck-Str. 4, 06120 Halle (Saale), Germany; frank.erdmann@pharmazie.uni-halle.de; 4 Department of Biology and Human Genetics, Faculty of Medicine, University of Niš, 18000 Niš, Serbia; s.sanja88@gmail.com (S.S.); stevo.najman@gmail.com (S.N.); 5 Department for Cell and Tissue Engineering, Scientific Research Center for Biomedicine, Faculty of Medicine, University of Niš, 18000 Niš, Serbia; 6 Laboratory of Biomedical Nanotechnologies, Institute of Bionic Technologies and Engineering, I.M. Sechenov First Moscow State University, Trubetskaya street 8, 119991 Moscow, Russia

**Keywords:** inflammation, glycosaminoglycans, LbL technique, macrophages adhesion, multinucleated giant cell (MNGCs) formation, NF-κB, immunoblotting, endocytosis

## Abstract

The use of implants can be hampered by chronic inflammatory reactions, which may result in failure of the implanted device. To prevent such an outcome, the present study examines the anti-inflammatory properties of surface coatings made of either hyaluronic acid (HA) or heparin (Hep) in combination with chitosan (Chi) prepared as multilayers through the layer-by-layer (LbL) technique. The properties of glycosaminoglycan (GAG)-modified surfaces were characterized in terms of surface topography, thickness and wettability. Results showed a higher thickness and hydrophilicity after multilayer formation compared to poly (ethylene imine) control samples. Moreover, multilayers containing either HA or Hep dampened the inflammatory response visible by reduced adhesion, formation of multinucleated giant cells (MNGCs) and IL-1β release, which was studied using THP-1 derived macrophages. Furthermore, investigations regarding the mechanism of anti-inflammatory activity of GAG were focused on nuclear transcription factor-кB (NF-κB)-related signal transduction. Immunofluorescence staining of the p65 subunit of NF-κB and immunoblotting were performed that showed a significant decrease in NF-κB level in macrophages on GAG-based multilayers. Additionally, the association of FITC-labelled GAG was evaluated by confocal laser scanning microscopy and flow cytometry showing that macrophages were able to associate with and take up HA and Hep. Overall, the Hep-based multilayers demonstrated the most suppressive effect making this system most promising to control macrophage activation after implantation of medical devices. The results provide an insight on the anti-inflammatory effects of GAG not only based on their physicochemical properties, but also related to their mechanism of action toward NF-κB signal transduction.

## 1. Introduction

Biomaterial implants can trigger an undesired host response upon surgical insertion in the human body leading to limited functionality, longevity and eventually to failure of the biomedical device [1,2]. Therefore, it is desirable to design biomaterials that will guide the inflammation process to achieve the desired function of the device. The series of events that will determine implant fate is initiated by adsorption of blood proteins and the recruitment of leukocytes. Monocytes represent key players at the implantation site, where they differentiate to macrophages [3,4] that secret pro-inflammatory cytokines such as IL-1β and tumour necrosis factor-α (TNFα), chemokines and growth factors [5,6]. Furthermore, the surrounding tissue may also be damaged by the biocide activity of reactive oxygen and nitrogen species secreted by macrophages [7]. The persistent stimulation of the immune system may shift acute to chronic inflammation, which is characterized by the formation of multinucleated giant cells (MNGCs) as a result of macrophage fusion in an attempt to phagocyte biomaterials larger in size than a single cell [8,9]. Eventually, fibroblasts are recruited upon the prolonged inflammatory phase, which may finally result in encapsulation of the biomedical device causing its failure [10,11].

A passive mechanism to control inflammation after implantation is based on making biomaterial surfaces hydrophilic or to exploit steric repulsion to reduce the opsonization of the implant by plasma proteins, which is achieved for example by covalent immobilisation of polyethylene glycol [12]. This requires often an activation of the biomaterial surface by chemical or plasma treatment [13]. However, the effect is often limited due to the lack of the ability to inhibit protein adsorption completely and for longer periods of time [14,15]. Another approach is the development of controlled-release systems of nonsteroidal anti-inflammatory drugs (NSAIDs) to reduce the inflammatory response, which can be achieved by blending polymers with NSAID or immobilisation of NSAID nanoparticles on biomaterial surfaces [16,17]. A non-covalent modification of implant materials can be achieved by building up multilayer systems using the layer-by-layer (LbL) method [18]. The LbL method is based on the alternating deposition of oppositely charged polyelectrolytes among them also biopolymers like chitosan (Chi), hyaluronan (HA), heparin (Hep) and others [19,20]. The obvious advantage of LbL is that no chemical or plasma activation of implants is required and that a broad range of molecules is available. Moreover, physical surface properties like wettability can be tuned to achieve hydrophilic surfaces like those based on Chi as polycation and Hep and HA as polyanions that adsorb lower quantities of proteins [21,22]. It is interesting to note that beside their hydrophilic nature the glycosaminoglycans (GAG) HA and Hep possess anti-inflammatory properties through their effect on the nuclear transcription factor-кB (NF-κB) signalling pathway, which makes them interesting candidates for regulation of inflammatory processes [23,24].

The NF-κB transcription factor family consists of a variety of homodimers and heterodimers that regulate and play a crucial rule in apoptosis, cell proliferation, differentiation, cell migration, inflammation as well as angiogenesis and metastasis [25,26]. The activation of NF-κB involves two major signalling pathways, the canonical (classic) and non-canonical (alternative) that depends on different stimuli and responding proteins [27]. The NF-κB transcription factor is a key factor in inflammation having a direct influence on the regulation of cytokine release as well as inflammatory-related gene activation and gene expression [26,28]. The anti-inflammatory properties of high molecular weight HA (HMW-HA) is achieved by its binding to cell receptor CD44 leading to down regulation of toll-like-receptor (TLR) signalling, which mediates NF-κB activation [29,30]. In addition, the binding of HA to CD44 promotes the release of anti-inflammatory cytokines like IL-2 and IL-10 [24,31]. On the other hand, Hep has two different mechanisms of inhibition on the NF-κB signalling pathway in which one is focused on inhibiting the translocation of the transcription factor into the nucleus. The second is explained as the ability of Hep to interfere non-specifically with the binding of NF-κB to DNA in the nucleus [32]. Hence, leukocyte adhesion and activation as well as pro-inflammatory cytokine production is downregulated as a result of the inhibitory effect of Hep toward the NF-κB signalling [33].

**Figure 1:**
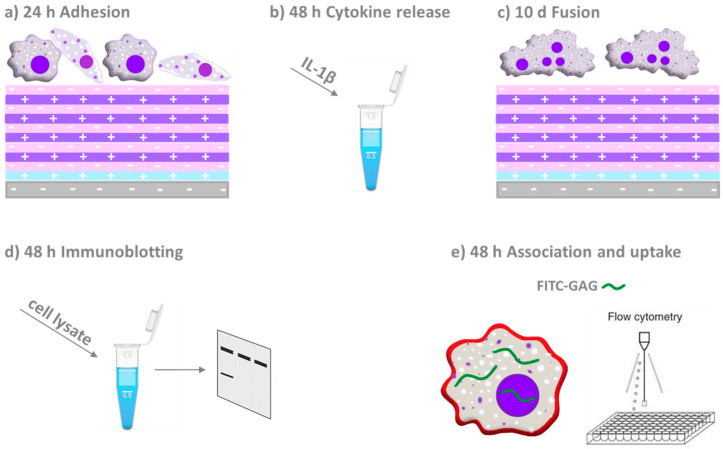
Schematic overview on the material system and design of the biological studies. Polyelectrolyte multilayers were assembled on model substrata glass or silicone (grey base layer with negative charge) on which a priming layer of poly (ethylene imine; blue layer with positive charge) was adsorbed first. Then alternatingly the polyanions heparin or hyaluronan (pink layer with negative charge) and chitosan as polycation (purple layer with positive charge) were adsorbed until 10 layers in total were assembled. Macrophages derived from the THP-1 monocytic cell line were seeded on these multilayers to study (**a**) adhesion and spreading of cells after 24 h, (**b**) evaluation of the pro-inflammatory (IL-1β) cytokine release, (**c**) multinucleated giant cells formation, (**d**) immunoblotting and confocal laser scanning microscopy (CLSM) to study the p65 subunit of NF-κB and (**e**) association and uptake of fluorescein isothiocyanate (FITC)-labelled GAG through macrophages by CLSM and flow cytometry.

In previous studies, we could show that covalent or adsorptive binding of HA and Hep make surface coatings that reduce macrophage adhesion and activation [21,34]. Here, we extend our findings shedding light on the underlying mechanism of action of multilayers made of Chi and HA or Hep focussing on the inhibition of the p65 subunit of NF-κB protein family as a target of canonical NF-κB pathway in THP-1 monocyte-derived macrophages. A survey on the general structure of polyelectrolyte multilayers (PEMs) and their effect on macrophages investigated by different biological studies are shown in Figure 1.

## 2. Results

### 2.1. Characterization of Physical Properties of Coatings

**Figure 2:**
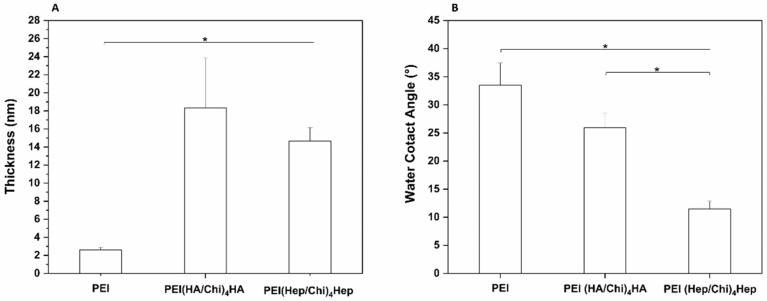
(**A**) Ellipsometry measurements to obtain the average thickness of poly (ethylene imine) (PEI) coating and multilayers made of either hyaluronic acid (HA) or heparin Hep as polyanions and chitosan (Chi) as a polycation, abbreviated as (PEI, PEI(HA/Chi)_4_HA and PEI(Hep/Chi)_4_Hep), respectively. Results represent means ± SD, *n* = 6, * *p* < 0.05. (**B**) Static water contact angle measurements using the sessile drop method to characterize surface wettability of the same surface coatings. Results represent means ± SD, *n* = 10, * *p* < 0.05.

The thickness of surface coatings prepared on silicon wafers was studied by ellipsometry in the dry state to verify the deposition of polyelectrolytes. The measurements were performed for the primary poly (ethylene imine) (PEI) layer that was used to provide a positive surface charge for binding the polyanions HA and Hep and formation of PEMs. The PEMs made of hyaluronan and chitosan were then designated as PEI(HA/Chi)_4_HA. The PEMs made of heparin and chitosan were abbreviated as PEI(Hep/Chi)_4_Hep. Altogether, 10 single layers were absorbed subsequently as described in the Materials and Methods Section. In Figure 2A a significantly higher thickness of PEMs was visible in comparison to PEI-modified silicon wafers. Static water contact angle (WCA) measurements were done to identify the wetting properties of the surface coatings. Figure 2B depicted a significantly lower WCA of PEMs compared to PEI surfaces. In addition, a significant difference between the two glycosaminoglycans was observed, showing the lowest WCA for multilayers composed of PEI(Hep/Chi)_4_Hep.

**Figure 3:**
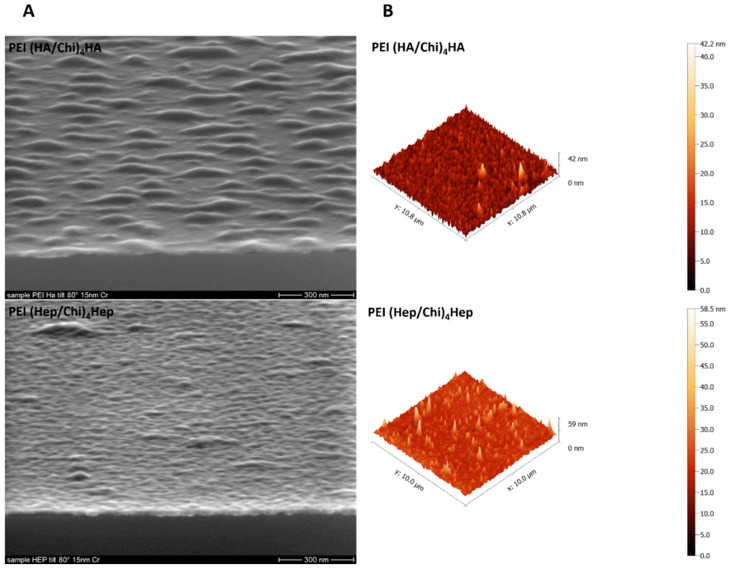
(**A**) Scanning electron microscopy (SEM), Scale bar: 300 nm and (**B**) atomic force microscopy (AFM) for studying topography of samples poly (ethylene imine) (PEI) and terminal layers of polyelectrolyte multilayers (PEMs) composed of either hyaluronic acid (HA) or heparin (Hep) as polyanions and chitosan (Chi) as polycation abbreviated as (PEI(HA/Chi)_4_HA, PEI(Hep/Chi)_4_Hep), respectively.

A deposition of a 15 nm Cr layer to achieve a sufficient conductivity of samples was performed prior to surface topography visualization with scanning electron microscopy shown in Figure 3A. PEMs containing HA demonstrated island-like structures while PEMs containing Hep expressed a more homogenous, smooth surface coverage. On the other hand, atomic force microscopy studies of surface topography shown in Figure 3B indicated smaller differences between both PEM, since the observed surface features had a similar range of 40–60 nm in the z scale though PEMs with HA as a terminal layer looked more homogenous here than those with Hep as a polyanion.

### 2.2. Adhesion of Macrophages and Multinucleated Giant Cell Formation

**Figure 4:**
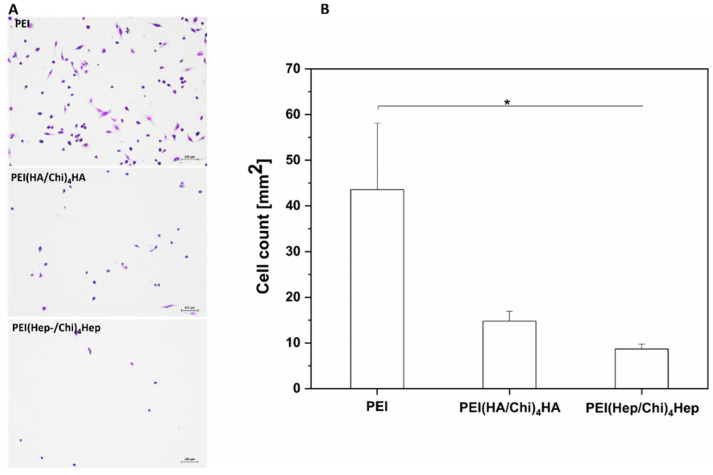
(**A**) Transmitted light microscopy images of adherent macrophages stained with 10% (v/v) Giemsa after 24 h on poly (ethylene imine) (PEI) and terminal layers of PEMs composed of either hyaluronic acid (HA) or heparin (Hep) as polyanions and chitosan (Chi) as polycation abbreviated as (PEI(HA/Chi)_4_HA, PEI(Hep/Chi)_4_Hep), respectively. Scale: 100 μm. (**B**) Number of adherent macrophages per surface area after 24 h of cultivation. Data represent means ± SD, *n* = 5, * *p* ≤ 0.05.

Micrographs visualizing the adhesion and shape of macrophages after 24 h of culture are shown in Figure 4A. Cells showed the highest adherence on PEI with a spread and elongated phenotype. On the other hand, a smaller number of predominantly round, less elongated macrophages were observed on PEMs. Quantitative data based on image analysis shown in Figure 4B displayed that the number of adherent macrophages was highest on the control substratum PEI, while the number of cells was significantly lower on PEMs with the smallest number on PEI(Hep/Chi)_4_Hep.

**Figure 5:**
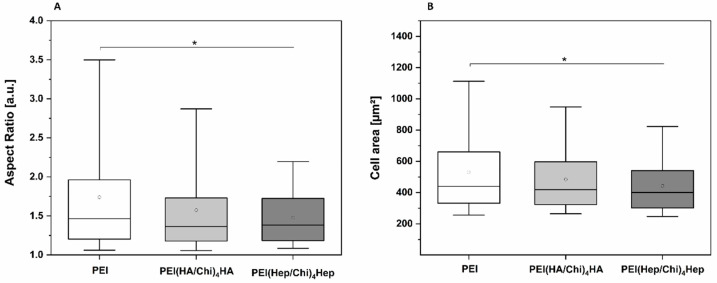
(**A**) Aspect ratio of adherent macrophages on poly (ethylene imine) (PEI) and terminal layers of PEMs composed of either hyaluronic acid (HA) or heparin (Hep) as polyanions and chitosan (Chi) as polycation abbreviated as (PEI(HA/Chi)_4_HA, PEI(Hep/Chi)_4_Hep), respectively. (**B**) Cell area of adherent macrophages per surface area after 24 h of cultivation. The box plot indicates the 25th and 75th percentile; the lowest and highest values are represented by the whiskers, whereas the median (dash) and mean value (white circle) are shown as well. *n* = 15, * *p* ≤ 0.05.

Image analysis was also used to quantify the size and shape of adherent macrophages. Figure 5A shows that the aspect ratio of adherent macrophages was higher related to an enhanced polarization of macrophages on PEI samples compared to cells on PEMs, where it was significantly lower. Figure 5B shows that also spreading of macrophages was significantly lower on PEMs in comparison to PEI.

**Figure 6:**
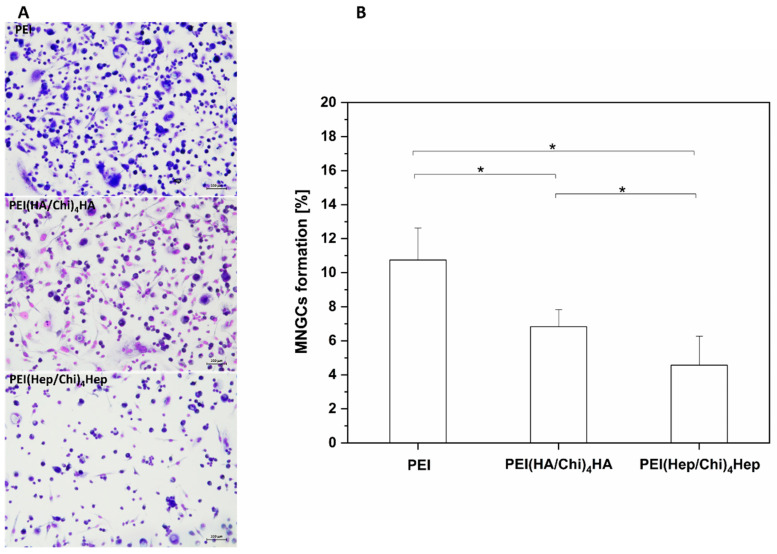
(**A**) Images of multinucleated giant cells (MNGCs) stained with 10% (v/v) Giemsa after 10 days cultivation on poly (ethylene imine) (PEI) and terminal layers of PEMs composed of either hyaluronic acid (HA) or heparin (Hep) as polyanions and chitosan (Chi) as polycation abbreviated as (PEI(HA/Chi)_4_HA, PEI(Hep/Chi)_4_Hep), respectively. Scale bar: 100 μm. (**B**) The area percentage of MNGCs on PEI, PEI(HA/Chi)_4_HA and PEI(Hep/Chi)_4_Hep surfaces based on quantitative image analysis of micrographs. Results represent means ± SD, * *p* < 0.05, *n* = 15.

Furthermore, micrographs presented in Figure 6A visualized significantly higher numbers of multinucleated giant cells (MNGCs) on PEI samples, which can be identified by the number of nuclei (≥2) per cell body as well as the larger cell size. By contrast, on PEMs a lower number of MNGCs was seen. The quantitative analysis of area percentage of MNGCs presented in Figure 6B shows that fusion of macrophages was significantly lower on PEMs in comparison to PEI. In addition, the formation of MNGCs was also significantly lower on PEI(Hep/Chi)_4_Hep in comparison to PEI(HA/Chi)_4_HA.

### 2.3. IL-1β Pro-Inflammatory Cytokine Release

**Figure 7:**
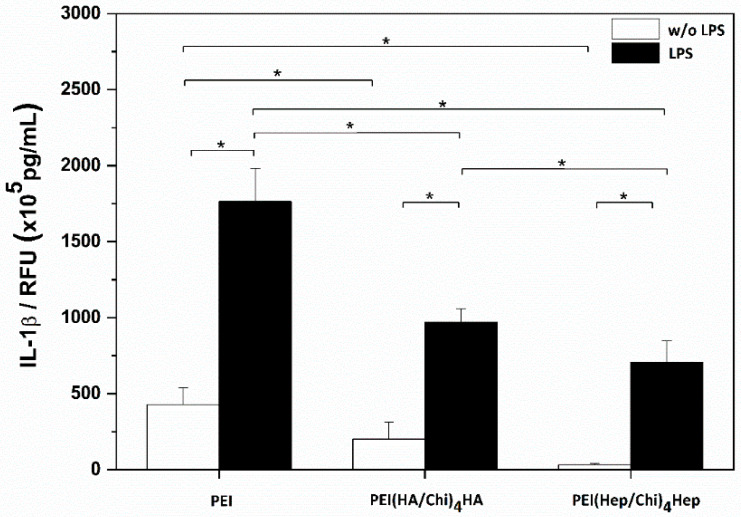
IL-1β release from macrophages after 24 h incubation in absence (white bars) and presence (black bars) of lipopolysaccharide (LPS) on poly (ethylene imine) (PEI) and terminal layers of PEMs composed of either hyaluronic acid (HA) or heparin (Hep) as polyanions and chitosan (Chi) as polycation abbreviated as (PEI(HA/Chi)_4_HA and PEI(Hep/Chi)_4_Hep), respectively. Data represent means ± SD, *n* = 6, * *p* ≤ 0.05.

Results of studies on the release of Interleukin-Iβ are shown in Figure 7. Two sets of samples were studied with the absence (white bars) and presence (black bars) of lipopolysaccharide (LPS). LPS stimulation leads to an up regulation of IL-1β in THP-1 derived macrophages, which is also an indicator of the functionality of these cells. Macrophages adhering on PEI-coated surfaces produced the highest quantity of IL-1β under both conditions (with or without LPS). By contrast macrophages cultured on both PEMs had a significantly reduced release of this cytokine in the presence and absence of LPS. In addition, IL-1β release from macrophages cultured on PEI(Hep/Chi)_4_Hep was significantly lower in comparison to PEI(HA/Chi)_4_HA.

### 2.4. Immunofluorescence Staining of NF-κB in Macrophages

**Figure 8:**
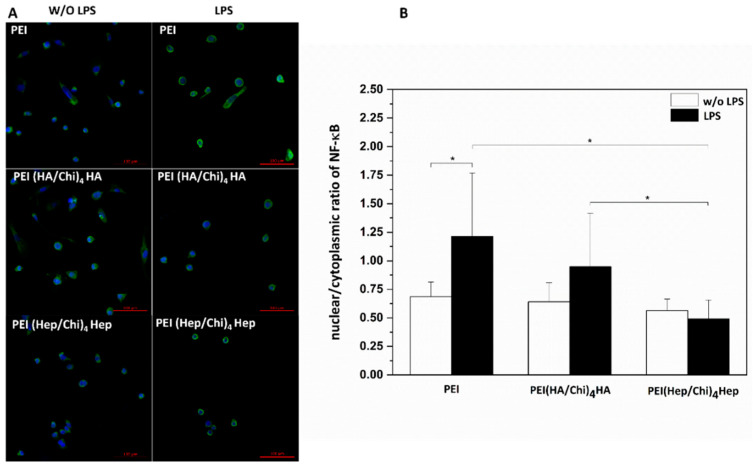
(**A**) TO-PRO-3 (blue colour) staining of nuclei and monoclonal antibody detection (green colour) of NF-κB p65 subunit shown for non-stimulated (left row) and stimulated (1 µg·mL^−1^ LPS) macrophages (right row). The cells were cultured for 48 h on poly (ethylene imine) (PEI) and terminal layers of PEMs composed of either hyaluronic acid (HA) or heparin (Hep) as polyanions and chitosan (Chi) as polycation abbreviated as (PEI(HA/Chi)_4_HA, PEI(Hep/Chi)_4_Hep), respectively. Scale bar: 100 μm. (**B**) Quantification of nuclear/cytoplasmic ratio in absence (white bars) and presence (black bars) of LPS in cells cultured on PEI and PEMs. Data represent means ± SD, *n* = 10, * *p* ≤ 0.05.

In Figure 8A the cell nuclei have been stained with the nuclear stain TO-PRO-3 (blue colour) and the non-phosphorylated p65 subunit of NF-κB with a monoclonal antibody (green colour). It is visible that the p65 subunit of NF-κB can be found both in the cell cytoplasm and nuclei. The nuclear to cytoplasmic ratio of NF-κB was quantitatively evaluated and used as an indicator for the translocation of the transcription factor into the nuclear area. The highest extent of p65 translocation was observed in macrophages on PEI; both in the presence and absence of LPS (Figure 8B). By contrast, a significantly lower nuclear to cytoplasmic ratio was found in macrophages cultured on PEMs. Indeed, the lowest quantity of p65 translocation into the nuclear area both in the absence and presence of LPS was found in cells cultured on PEI(Hep/Chi)_4_Hep (Figure 8B).

### 2.5. Western Blotting

**Figure 9:**
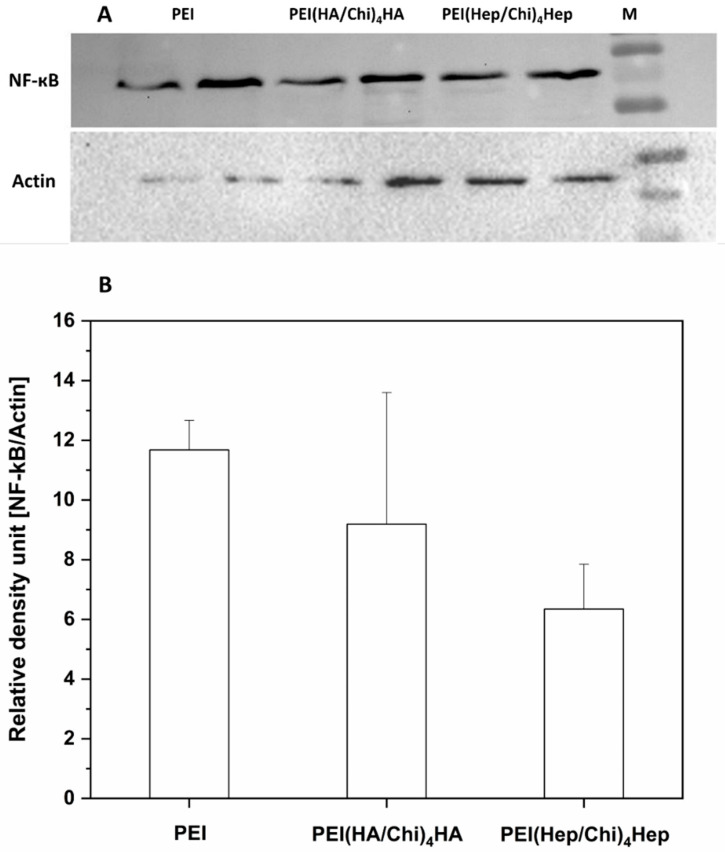
(**A**) Western blots with bands of p65 of NF-κB and actin from two samples of lysates of macrophages cultured on poly (ethylene imine) (PEI) and terminal layers of PEMs composed of either hyaluronic acid (HA) or heparin (Hep) as polyanions and chitosan (Chi) as a polycation abbreviated as (PEI(HA/Chi)_4_HA, PEI(Hep/Chi)_4_Hep), respectively. The lysates, collected after 48 h, were blotted toward (NF-κB) and actin. (**B**) The immunoblotting bands were analysed by densitometry. Bands of p65 subunit of NF-κB were normalized to expression of actin. The ratio was named as relative density units. Data represent means, *n* = 2.

Figure 9A depicts the bands for the non-phosphorylated p65 subunit of NF-κB (named here as NF-κB only) and actin, in which the latter was used for normalization of data in the quantitative evaluation by densitometry. It is visible that a higher expression of NF-κB was observed in cell lysates from macrophages cultured on PEI samples compared to cells on PEMs. Figure 9B shows the quantitative evaluation of band intensities of NF-κB expression in macrophages cultured on PEI and PEMs, quantified by densitometry. The lowest intensity of NF-κB in cell lysates was observed in macrophages cultured on PEI(Hep/Chi)_4_Hep. The original blots from different gels can be found in Figures S2 and S3.

### 2.6. Association of GAG with Macrophages Studied by Confocal Laser Scanning Microscopy (CLSM)

**Figure 10:**
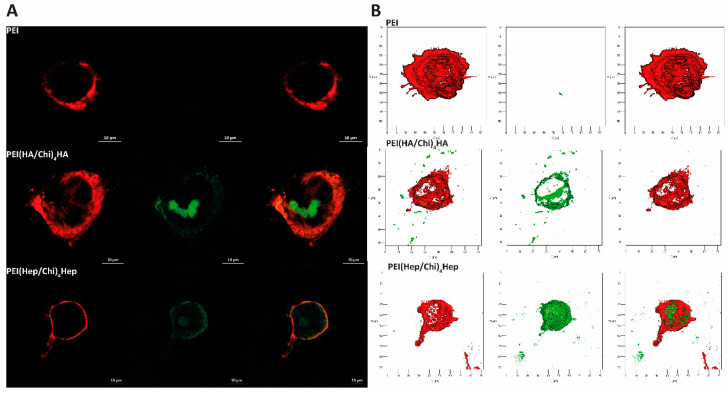
(**A**) Representative confocal laser microscopy (CLSM) images of adherent macrophages stained with the membrane stain DID (red colour) after 24 h cultivation on poly (ethylene imine) (PEI) and terminal layers of PEMs composed of either hyaluronic acid (HA) or heparin (Hep) as polyanions (stained with FITC, green colour) and chitosan (Chi) as a polycation abbreviated as (PEI(HA/Chi)_4_HA, PEI(Hep/Chi)_4_Hep), respectively (63-fold oil immersion objective, Scale bar: 10 µm). (**B**) Representative 3D view of a z-stacks in surface projection with CLSM (63-fold oil immersion objective, scale: 20 μm). In this mode, pixel values are computed as solids, which allows no transparency.

DID-stained macrophages cultured on terminal layers of PEMs containing fluorescent FITC-labelled glycosaminoglycans (GAG, green colour) visualized by CLSM are shown in Figure 10. Here, the macrophages cultured on PEI expressed a red staining of DID of the cell membrane, only because no FITC-labelled GAG were present on this surface (Figure 10A). The macrophages cultured on PEMs with FITC-labelled GAG show an association of FITC-labelled HA or Hep (green in confocal images) with DID-stained (red) cells. 3D images of FITC-labelled HA and Hep are shown in Figure 10B. Here, it is visible that HA or Hep were either co-localized with the cell surface or found intracellularly. Figure 10A,B shows the ability of macrophages to associate with and the uptake of the immobilized FITC-GAG. Additional cell images are provided in Figures S4 and S5.

### 2.7. Association of FITC-Labelled GAG with Macrophages Studied by Flow Cytometry

**Figure 11:**
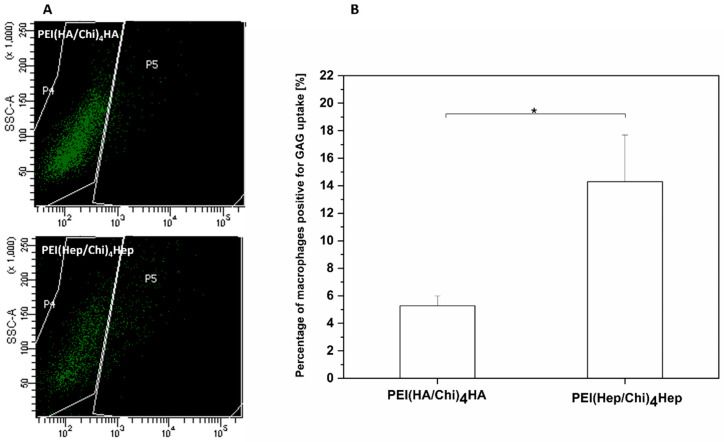
(**A**) Illustrative images of the flow cytometry measurements of GAG association and uptake by macrophages cultured on either FITC-labelled hyaluronic acid or FITC-heparin multilayers abbreviated as (PEI(HA/Chi)_4_HA, PEI(Hep/Chi)_4_Hep), respectively. (**B**) The percentage of macrophages positive for GAG uptake after 48 h cultivation on PEI(HA/Chi)_4_HA and PEI(Hep/Chi)_4_Hep. Data represent means ± SD, *n* = 4, * *p* ≤ 0.05.

The results of flow cytometry with dot blots of the side scatter (SSC, y axis) versus the FITC fluorescence (x axis) are shown in Figure 11A. Macrophages located in the P4 region were considered to be negative for GAG-FITC due to cell auto-fluorescence. An uptake of FITC-labelled-GAG by macrophages is denoted by an increase of the cellular fluorescence related to the emission of fluorescein (FITC). Hence, the P5 region shows the number of macrophages positive for FITC-labelled-GAG. Figure 11B shows the quantitative evaluation of macrophages positive for FITC-GAG demonstrating that cells cultured on multilayers containing HA expressed a significantly lower uptake of FITC-labelled GAG in comparison to the cell cultured on PEM with FITC-heparin.

## 3. Discussion

This study aimed to investigate the mechanism of the anti-inflammatory action of PEMs systems based on either HA or Hep as polyanions in combination with Chi as a polycation fabricated by the LbL technique to explore their potential for making implantable biomedical devices more immune compatible to avoid chronic inflammation and subsequent fibrosis. The polyethylene imine (PEI) that serves often as an anchoring layer in the LbL technique [35] was used here for comparison because it is known that amino-terminated surfaces provoke an activation of macrophages [36], which is also known for PEI-modified substrata [34].

Physicochemical studies were performed to characterize surface properties like wettability and topography that have an impact on protein adsorption and cell adhesion [37]. Studies of the topography and thickness showed that the LbL technique was able to achieve a complete coating of substrata (glass or silicone) with PEMs of a dry thickness in the range of 15–20 nm with a rather smooth surface topography in the micrometre scale with differences between HA and Hep multilayer systems. Indeed, multilayers with HA showed a more island-like structure when studied with SEM, which might be related to the much larger molecular weight of hyaluronan compared to heparin used as polyanions in multilayer formation. Wettability studies showed that both glycosaminoglycans HA and Hep formed multilayer coatings of more hydrophilic character despite the presence of chitosan as found also in previous studies [38,39]. By contrast, highest WCA was found for the PEI modified surface, which also corresponds typically to increased protein adsorption, adhesion, and spreading of cells in comparison to more hydrophilic substrata [34].

Accordingly, the result of macrophage adhesion and spreading corresponded to the wetting properties of surfaces showing that it was highest on PEI, while PEMs with GAG reduced their adhesion and spreading with lowest on PEI(Hep/Chi)_4_Hep. However, macrophage adhesion was not statistically different between HA and Hep-terminated PEMs. In contrast to that MNGC formation was significantly decreasing from PEI to HA and then Hep. Although, MNGC formation is related to the number of adhering macrophages that are required to aggregate before their fusion, other factors like cytokine release play a more dominant role [40]. Therefore, studies on the release of IL-1β were performed that demonstrated a significant reduction of release of this pro-inflammatory cytokine from macrophages cultured on PEMs. It should be noted that IL-1β release was normalized to the number of viable cells. Hence, the reduced adhesion of macrophages due to higher wettability of PEM alone as a physical effect was not sufficient to explain the suppressive effect of multilayer coatings based on HA and Hep on macrophage activation.

Since, wetting properties of HA and Hep-based PEMs were quite similar, pharmaceutical effects of both GAG might play an important role for the observed inhibition on macrophage fusion and release of pro-inflammatory cytokines. It is known that reduced secretion of pro-inflammatory cytokines like IL-1β is related to the inhibition of NF-κB signalling pathway [41,42]. Hence, the focus was set on the study of the NF-κB signalling pathway, which plays a pivotal role during the activation of macrophages and other cells [26]. As it could be expected from the studies on macrophage adhesion, fusion and cytokine release, translocation of the p65 subunit of NF-κB to the nuclear area and its concentration were highest in macrophages on the control surface PEI. On the other hand, all parameters were lower in macrophages cultured on PEMs based on either HA or Hep. However, most effective in this regard was the multilayer system PEI(Hep/Chi)_4_Hep. A question how both GAG can control the activation of macrophages was related to the fact that both GAGs were physically bound to chitosan in multilayers. Indeed, we have shown previously that such multilayer systems made of biogenic polyelectrolytes represent a living interface, in which polyelectrolytes can interact with and be translocated by cells [43]. Hence, we studied association and potential internalization of FITC-labelled GAG by macrophages using CLSM showing that both association as well as internalization of these GAG can occur. Moreover, studies with flow cytometry confirmed such an association and showed also that a larger quantity of FITC-Hep was found to be associated with macrophages in comparison to FITC-HA.

Indeed, heparin can be internalized by cells either through anionic membrane transporters or by endocytosis [44]. It is known that heparin, taken up by endocytosis, may bind to the positively charged p50–p65 subunits of NF-κB, leading to a partial inhibition of the phosphorylation process and a reduced translocation of the transcription factor to the nucleus [44,45]. A further mechanism is that intracellular heparin can interfere non-specifically with DNA binding of NF-κB in the nucleus [32]. On the other hand, the high molecular weight form of hyaluronan (HMW-HA) used here for fabrication of PEMs possesses an anti-inflammatory potential through cross-linking the surface receptor CD44 on cells like macrophages, which suppresses the pro-inflammatory signaling by toll-like receptors (TLR), resulting in down-regulation the phosphorylation cascade of NF-κB pathway [46]. In addition, CD44 is thought to play an important role in the reduction of pro-inflammatory cytokines release by other pathways [13,47]. The intracellular presence of FITC-HA shown by CLSM may be related to CD44 by receptor endocytosis with the bound ligand [48], but probably not affecting intracellular signal transduction. Since, NF-κB signalling is one of the important pathways of the regulation of cytokine gene expression, an inhibition by both hyaluronan and heparin, may eventually decrease the potential inflammatory response of macrophages [49,50], which is obviously the case for both multilayer systems that have been presented in this study.

## 4. Materials and Methods

### 4.1. Chemicals for Surface Modification

Glass cover slips, Ø 12 mm and 15 mm, were provided from Menzel GmbH (Bielefeld, Germany). Silicon wafers of 10 × 10 mm^2^ surface were obtained from LG Siltron Inc. (Gumi, Korea). Poly (ethylene imine) (PEI, M*_w_* ≈ 750 kDa) was purchased from Polysciences Inc. (Warrington, PA, USA). Heparin (Hep, M*_w_* ≈ 15 kDa) and hyaluronic acid (HA, M*_w_* ≈ 1.3 MDa) were provided from Serva (Heidelberg, Germany) and Innovent e.V. (Jena, Germany), respectively. Labelling of GAG was done with fluorescein isothiocyanate (FITC) according to the protocol published recently to obtain 10% labelled carboxylic groups of either HA or Hep with FITC [51]. Chitosan 85/500 with a deacetylation degree of 85% (Chi, M*_w_* ≈ 500 kDa) was delivered from Heppe Medical Chitosan GmbH (Halle, Germany). Sodium chloride (NaCl) was obtained from Roth (Karlsruhe, Germany), while acetic acid was provided from Applichem (Darmstadt, Germany). Ammonia (25%) and hydrogen peroxide (30%) were purchased from TH-Geyer GmbH and Co. KG and Roth (Renningen, Karlsruhe, Germany), respectively. AFM tips were provided from AppNano (Applied Nanostructures Inc., Santa Clara, CA, USA).

### 4.2. Substrates and Polyelectrolyte (PEL) Preparation

The physicochemical and biological properties of multilayers were investigated by using model substrates, such as silicon wafers and glass cover slips. A solution of ammonia, hydrogen peroxide and water (1:1:5, v/v/v) at 75 °C for 10 min was used for cleaning of substrata. Thereafter, the wafers as well as the glass cover slips were washed with ultrapure water (6 × 5 min), and dried with a stream of nitrogen [52]. Hyaluronic acid, heparin and chitosan solutions were prepared at a concentration of (2 mg·mL^−1^) by dissolution in 150 mM NaCl at pH 4.0. Poly (ethylene imine) was dissolved at a concentration of 5 mg·mL^−1^ in 150 mM NaCl at pH 7.0 as in previous studies [17]. Poly (ether sulfone) filters of 0.2 µm pore size was used for sterilization of solutions.

### 4.3. Polyelectrolyte Multilayers (PEMs) Formation

An anchoring base layer of poly (ethylene imine) was applied to obtain a positive surface net charge on silicon and glass substrates for subsequent adsorption of polysaccharides as done in previous studies [17]. In addition, this substrate was used as a control for comparison with multilayers. PEI was adsorbed for 30 min on glass or silicone slides and rinsed with 150 mM NaCl at pH 7.0, three times for 5 min each. Subsequently, multilayers of HA or Hep as polyanions, followed by washing with PBS and subsequent adsorption of Chi for 15 min each until 4 double layers and a final GAG layer were obtained. The control surface was always abbreviated as PEI, while the multilayer systems were always abbreviated as either (PEI, PEI(HA-Chi)_4_HA or PEI(Hep-Chi)_4_Hep). In studies on association and uptake of FITC-GAG, the last two GAG layers consisted of either FITC-HA or FITC-Hep [17].

### 4.4. Characterization of Surface Properties and Multilayer Formation

#### 4.4.1. Scanning Electron Microscopy (SEM)

The coated silicon wafers with PEMs were analysed by Philips ESEM XL 30 FEG (Eindhoven, Netherlands) in high vacuum (*p* = 10^−6^ mbar) to visualize the surface topography. A conductive layer of 15 nm thick chromium (Cr) was deposited by sputtering.

#### 4.4.2. Atomic Force Microscopy (AFM)

AFM (Nano-R, Pacific Nanotechnology, Santa Clara, CA, USA) was also used to study surface topography of PEI- and GAG-modified silicon wafers (Si) in a three-dimensional view. A contact mode under ambient (air) laboratory conditions of temperature and humidity was selected in order to probe the coated Si wafers (10 × 10 mm^2^). Images were taken by using AFM tips with 125 µm length, 35 µm width, 14−16 µm height and a tip radius of <10 nm. A resolution of (512 × 512 pixel^2^) as well as a scan area of 10 × 10 μm^2^ per image was applied with a scan rate of 0.2 Hz. Gwiddyon software (Nano-R, Pacific Nanotechnology, Santa Clara, CA, USA) (version 2.40) was used for image processing [17].

#### 4.4.3. Water Contact Angle (WCA)

The wettability of the samples was determined with static water contact angle (WCA). An OCA 15+ device from Dataphysics (Filderstadt, Germany) using the sessile drop method was applied here. Ultrapure water of 2 μL with a minimum of five droplets was investigated to each sample (2 for each material) at room temperature. The obtained values were used to calculate the means and standard deviations [38].

#### 4.4.4. Measurement of Multilayer Thickness by Ellipsometry

The average thickness of the PEMs was determined by the spectroscopic ellipsometry (M–2000 V, J.A. Woollam Company, Lincoln, NE, USA). The used reference substrate was a cleaned Si wafer with a SiO_2_ layer thickness of 2.5 nm. A Cauchy model was used to extract the optical constants of the multilayers, which was previously described in the literature [51].

### 4.5. Studies with THP-1 Derived Macrophages

#### 4.5.1. Cell Culture

RPMI-1640 medium (Lonza, Wuppertal, Germany) supplemented with 10% (v/v) foetal bovine serum (FBS, Biochrom AG, Berlin, Germany) and 1% (v/v) antibiotic–antimycotic solution (AAS, Lonza, Wuppertal, Germany) was used for culturing THP-1 human monocytic cells (DSMZ, Braunschweig, Germany) at 37 °C in a humidified 5% CO2/95% air atmosphere in a NUAIRE^®^ DH Autoflow incubator (NuAire, Plymouth, MN, USA). Cells were passaged every second day to maintain a cell density of 1 × 10^6^ cells mL^−1^. Macrophages were differentiated from floating THP-1 cells by incubation with 200 nM phorbol-12-myristate-13-acetate (PMA, Sigma Aldrich, Darmstadt, Germany) in T75 cell culture flasks (Greiner Bio-One GmbH and Co.KG, Frickenhausen, Germany) for 48 h. Afterwards, 0.25% trypsin/0.02% EDTA (Biochrom AG, Berlin, Germany) was used to detach the adherent macrophages with further addition of serum-containing RPMI-1640 medium to stop the trypsin effect. Finally, the harvested cells were used for seeding on the different PEMs-modified surfaces [34].

#### 4.5.2. Cell Adhesion Studies

An ultraviolet light chamber (Bio–Link BLX, LTF Labortechnik GmbH and Co. KG, Wasserburg, Germany) set at 254 nm (50 J·cm^−2^) was used for sterilization of PEM modified samples and PEI coated control surfaces (samples were placed in 24-well tissue culture plates, Greiner Bio-One GmbH and Co.KG, Frickenhausen, Germany). Sterilization by UV light was done for 60 min prior to cell studies. Macrophages were seeded at a cell density of 2.5 × 10^4^ cells·mL^−1^ in serum-containing RPMI-1640 medium. Cells were cultured on the samples for 24 h at 37 °C in a humidified 5% CO_2_/95% air atmosphere. Thereafter, gentle washing with phosphate buffer saline (PBS) was done twice to remove non-adherent cells. The attached cells were fixed with cold methanol (Roth, (Karlsuhe, Germany)) for 10 min and stained with 10% (v/v) Giemsa (Merck KGaA, Darmstadt, Germany) in ultrapure water for another 10 min. Micrographs were taken by a light microscope (Nikon ECLIPSE Ti2, Tokyo, Japan) equipped with a CMOS camera (Nikon DS-Fi3, Tokyo, Japan). ImageJ software (version 1.52p, https://imagej.nih.gov) was used to quantify the number of adhering cells of the images [21].

#### 4.5.3. Analysis of Multinucleated Giant Cells (MNGCs) Formation

The formation of MNGCs was evaluated through MNGCs area percentage after culturing macrophages, initially seeded at density of 2.5 × 10^5^ cells·mL^−1^, on the PEMs and PEI surfaces for 10 days. Samples were gently washed twice with PBS followed by fixation of attached cells with cold methanol and staining with 10% (v/v) Giemsa in ultrapure water. Cells were imaged using light microscopy. The area percentage of MNGCs was calculated by ImageJ software [38].

#### 4.5.4. IL-1β Production Measurement

The pro-inflammatory cytokine release was investigated using an enzyme linked immunosorbent assay (ELISA). The measurements were performed according to the manufacturer’s instructions (Thermo Scientific, Germany) for the medium supernatants of samples. Two sets of samples were collected after 24 h of incubation in the absence and presence of LPS and stored at −20 °C until needed for investigation. A QBlue^®^ cell viability assay (BioChain, California, USA) was used to estimate the cell viability in attempt to normalize the cytokine production to the quantity of metabolic active cells on the different PEMs surfaces.

Therefore, the attached cells of the different surfaces were washed carefully with sterile PBS after supernatant collection. Then, a pre-warmed, colourless Dulbecco’s modified Eagle’s medium (DMEM) with QBlue^®^ assay reagent (10:1) were added and incubated for 2 h at 37 °C in humidified 5% CO_2_/95% air atmosphere. Eventually, the relative fluorescence unit (RFU) values were measured after transferring 100 μL of the supernatant from each well to a black 96-well plate. The values were measured at an excitation wavelength of 544 nm and emission wavelength of 590 nm with plate reader [34].

#### 4.5.5. Immunofluorescence (IF) Staining of NF-kB

Immunostaining was performed to study the translocation of the p65 subunit of NF-κB according to the method developed by Noursadeghi et al. [53]. Macrophages were seeded like in the aforementioned section and cultured for 48 h. Samples were analysed in two sets in the absence and presence of 1 µg·mL^−1^ lipopolysaccharide (LPS, Sigma Aldrich, Darmstadt, Germany). Thereafter, fixation of the cultured cells on PEI and PEMs surfaces was performed with 4% paraformaldehyde (Sigma Aldrich, Darmstadt, Germany) for 15 min, permeabilized with 0.1% (v/v) Triton^®^ X-100 (Sigma-Aldrich, Taufkirchen, Germany) for 10 min at RT, and rinsed twice with PBS. The non-specific binding sites were blocked by using bovine serum albumin (BSA, ≥98%, Carl Roth GmbH, Halle (Saale), Germany; 1%, w/v) in PBS for 30 min. Afterwards, cells were incubated with a monoclonal p65 subunit of the NF-κB antibody (1:100, Santa Cruz Biotechnology, Dallas, TX, USA) at 4 °C overnight. A secondary monoclonal anti-rabbit IgG antibody conjugated with CY2 (1:200, Jackson Immunoresearch, Ely, UK) was applied for another 30 min at RT after washing with PBS for 5 min on a shaker. TO-PRO-3 (1:500, Invitrogen, CA, USA) for 40 min at RT was used for the nuclei staining. Eventually, confocal microscope LSM 710 (Carl Zeiss, Oberkochen, Germany) applying a 40-fold oil immersion objective was utilized to examine the samples that were mounted on glass slides with polyvinyl alcohol (PVA, Sigma Aldrich, Darmstadt, Germany). ImageJ (v.1.52i) software was used for image processing. The TO-PRO-3 channel was used to mask a region of interest (ROI). Then the nuclear ROI was subtracted from the cellular ROI to obtain a cytosolic ROI. Finally, the fluorescence intensity was evaluated in the nuclear and cytosolic ROI and a ratio calculated [21]. The principle of the method is visualized in Figure S1.

#### 4.5.6. Cell Lysis for Immunoblotting (IB)

THP-1 derived macrophages were differentiated as described above and cultured for 48 h at a cell density of 52 ×10^5^ cells·mL^−1^ on the prepared PEMs and PEI on objective glass slides with a total area of 19.76 cm^2^ (Menzel GmbH, Bielefeld, Germany) in the four well plates (Greiner Bio-one, Leipzig, Germany). Thereafter, plates were placed on ice and the cells were washed twice with ice-cold PBS. Afterwards, the macrophages were scraped using a cold plastic cell scraper after adding a cell lysate buffer (RIPA buffer) with protease and phosphatase inhibitors (Thermo Fisher scientific, Waltham, MA, USA). Then, a constant agitation at 4 °C for 30 min was maintained beyond the gentle transfer of cell lysates into pre-cooled tubes. Subsequently, the cell lysates were centrifuged at 4 °C and 13,000 rpm for 20 min then the supernatants were stored at −80 °C up to one month before performance of IB [21].

#### 4.5.7. SDS-PAGE and Western Blotting

The same amount of proteins from each samples cell lysate were separated in 15% SDS-polyacrylamide gels and transferred onto nitrocellulose membranes (Schleicher and Schuell GmbH, Munich, Germany). In TBS-T, 3% (w/v) milk powder was used to block membranes, probed with monoclonal antibodies against phospho-NF-κB (CST, #3033), NF-κB (Santa Cruz Biotechnology, sc-8008) and β-actin (MERCK, A1978), followed by incubation with HRP-labelled secondary antibodies and visualized with the enhanced chemiluminescence reaction (Thermo Fisher Scientific, Waltham, MA, USA) in a ChemiDoc Imaging System (Bio-Rad, CA, USA). Obtained grayscale images were densitometrically analysed by Li-Cor Image Studio software using automatic background determination and subtraction function [21].

#### 4.5.8. Association of FITC-GAG with Macrophages Studied by Confocal Laser Scanning Microscopy

The same density as in the MNGCs formation analysis was used for culturing macrophages for 24 h on PEI and PEMs with terminal layers of FITC-labelled GAG. Cell fixation was performed by adding 4% paraformaldehyde (Sigma Aldrich, Darmstadt, Germany) for 10 min. Thereafter, each sample and incubated for 10 min after adding 0.5 mL of membrane dye carbocyanine (DID, Biotium, Fremont, CA, USA) solution (5 µL of dye in 1 mL PBS). Eventually, CLSM using 63-fold oil immersion objective (LSM 710, Carl Zeiss, Oberkochen, Germany) was used to examine all samples that were washed twice with PBS, mounted on objective slides using polyvinyl alcohol (PVA, Sigma Aldrich, Darmstadt, Germany). ZEN2011 software (Carl Zeiss, Oberkochen, Germany) was utilized for image processing [21].

#### 4.5.9. Uptake of FITC-GAG by Macrophages Studied with Flow Cytometry

Cells were seeded on PEI and PEMs with the terminal two single layers of FITC-labelled GAG and cultured for 48 h as described in the aforementioned section of CLSM. The cells were scraped after trypsinization, centrifuged, washed once with PBS and resuspended in 200 µL PBS. Then, a flow cytometry device (LSR Fortessa II, BD Bioscience, Germany) was used to measure the 100 µL cell suspension, which was transferred to 96-well plate. FACS-Diva software (LSR Fortessa II, BD Bioscience, Germany) (version 6.2) was used for data analysis [21].

### 4.6. Statistics

An origin 8 Pro software (Origin Lab, Northampton, MA, USA) was used here for the statistical calculations. The one-way analysis of variance (ANOVA) followed by post-hoc Tukey’s test was applied. The mean values ± standard deviations (SD) represented all used data. It is indicated in the respective figure captions, the samples’ number. Statistical significance was considered for *p* ≤ 0.05 and is visualized by asterisks in the figures.

## 5. Conclusions

In this study, it was shown that the anti-inflammatory effect of PEMs made of either heparin or hyaluronan with chitosan as a polycation were related to reduced adhesion, interleukin I-β release and macrophage fusion, when compared to a pro-inflammatory model surface based on the highly cationic polyethylene imine. It was evident that the observed effects are not solely based on the hydrophilic character of these PEMs when compared to PEI. Indeed, it was shown that the pharmaceutical effects of heparin and hyaluronan known from other studies came into play when macrophages adhering on PEMs associated with and took up these molecules through different membrane receptors that may lead to the suppression of the canonical NF-κB signalling pathway. Hence, such multilayer systems may be of great interest and potential to modulate inflammatory responses of biomaterials improving function and lifetime of implantable biomedical devices.

## Supplementary Materials

Supplementary materials can be found at https://www.mdpi.com/1422-0067/21/10/3724/s1.

## References

[1] Tang L., Eaton J.W. (1995). Inflammatory responses to biomaterials. Am. J. Clin. Pathol..

[2] Anderson J.M. (2009). In vitro and in vivo monocyte, macrophage, foreign body giant cell, and lymphocyte interactions with biomaterials. Biological Interactions on Materials Surfaces.

[3] Mariani E., Lisignoli G., Borzì R.M., Pulsatelli L. (2019). Biomaterials: Foreign bodies or tuners for the immune response?. Int. J. Mol. Sci..

[4] Murray P.J., Wynn T.A. (2011). Protective and pathogenic functions of macrophage subsets. Nat. Rev. Immunol..

[5] Schutte R.J., Parisi-Amon A., Reichert W.M. (2009). Cytokine profiling using monocytes/macrophages cultured on common biomaterials with a range of surface chemistries. J. Biomed. Mater. Res..

[6] Wynn T.A., Barron L. (2010). Macrophages: Master regulators of inflammation and fibrosis. Semin. Liver Dis..

[7] Fialkow L., Wang Y., Downey G.P. (2007). Reactive oxygen and nitrogen species as signaling molecules regulating neutrophil function. Free Radic. Biol. Med..

[8] Underhill D.M., Goodridge H.S. (2012). Information processing during phagocytosis. Nat. Rev. Immunol..

[9] Xia Z., Triffitt J.T. (2006). A review on macrophage responses to biomaterials. Biomed. Mater..

[10] Serini G., Bochaton-Piallat M.-L., Ropraz P., Geinoz A., Borsi L., Zardi L., Gabbiani G. (1998). The fibronectin domain ED-A is crucial for myofibroblastic phenotype induction by transforming growth factor-β1. J. Cell Biol..

[11] Barron L., Wynn T.A. (2011). Fibrosis is regulated by Th2 and Th17 responses and by dynamic interactions between fibroblasts and macrophages. Am. J. Physiol. -Gastrointest. Liver Physiol..

[12] Ratner B.D., Bryant S.J. (2004). Biomaterials: Where we have been and where we are going. Annu. Rev. Biomed. Eng..

[13] Altman R.D., Manjoo A., Fierlinger A., Niazi F., Nicholls M. (2015). The mechanism of action for hyaluronic acid treatment in the osteoarthritic knee: A systematic review. Bmc Musculoskelet. Disord..

[14] Franz S., Rammelt S., Scharnweber D., Simon J.C. (2011). Immune responses to implants–a review of the implications for the design of immunomodulatory biomaterials. Biomaterials.

[15] Vroman L., Adams A., Fischer G., Munoz P. (1980). Interaction of high molecular weight kininogen, factor XII, and fibrinogen in plasma at interfaces. Blood.

[16] Suarez P., Rojo L., Gonzalez-Gomez A., Roman J.S. (2013). Self-assembling gradient copolymers of vinylimidazol and (acrylic)ibuprofen with anti-inflammatory and zinc chelating properties. Macromol. Biosci..

[17] Al-Khoury H., Espinosa-Cano E., Aguilar M.a.R., Romaán J.S., Syrowatka F., Schmidt G., Groth T. (2019). Anti-inflammatory Surface Coatings Based on Polyelectrolyte Multilayers of Heparin and Polycationic Nanoparticles of Naproxen-Bearing Polymeric Drugs. Biomacromolecules.

[18] Borges J., Mano J.F. (2014). Molecular interactions driving the layer-by-layer assembly of multilayers. Chem. Rev..

[19] Benkirane-Jessel N., Schwinte P., Falvey P., Darcy R., Haïkel Y., Schaaf P., Voegel J.C., Ogier J. (2004). Build-up of polypeptide multilayer coatings with anti-inflammatory properties based on the embedding of piroxicam–cyclodextrin complexes. Adv. Funct. Mater..

[20] Shao J., Wen C., Xuan M., Zhang H., Frueh J., Wan M., Gao L., He Q. (2017). Polyelectrolyte multilayer-cushioned fluid lipid bilayers: A parachute model. Phys. Chem. Chem. Phys..

[21] AlKhoury H., Hautmann A., Erdmann F., Zhou G., Stojanovic S., Najman S., Groth T. (2020). Study on the potential mechanism of anti-inflammatory activity of covalently immobilized hyaluronan and heparin. J. Biomed. Mater. Res..

[22] Aggarwal N., Altgärde N., Svedhem S., Michanetzis G., Missirlis Y., Groth T. (2013). Tuning Cell Adhesion and Growth on Biomimetic Polyelectrolyte Multilayers by Variation of p H During Layer-by-L ayer Assembly. Macromol. Biosci..

[23] Mehta V.B., Besner G.E. (2003). Inhibition of NF-κB activation and its target genes by heparin-binding epidermal growth factor-like growth factor. J. Immunol..

[24] Neumann A., Schinzel R., Palm D., Riederer P., Münch G. (1999). High molecular weight hyaluronic acid inhibits advanced glycation endproduct-induced NF-κB activation and cytokine expression. FEBS Lett..

[25] Karin M., Greten F.R. (2005). NF-κB: Linking inflammation and immunity to cancer development and progression. Nat. Rev. Immunol..

[26] Lawrence T. (2009). The nuclear factor NF-kappaB pathway in inflammation. Cold Spring Harb. Perspect Biol..

[27] Liu T., Zhang L., Joo D., Sun S.-C. (2017). NF-κB signaling in inflammation. Signal Transduct. Target. Ther..

[28] Ghosh S., Hayden M.S. (2008). New regulators of NF-κB in inflammation. Nat. Rev. Immunol..

[29] Avenoso A., D’Ascola A., Scuruchi M., Mandraffino G., Calatroni A., Saitta A., Campo S., Campo G.M. (2018). Hyaluronan in the experimental injury of the cartilage: Biochemical action and protective effects. Inflamm. Res..

[30] Altman R., Bedi A., Manjoo A., Niazi F., Shaw P., Mease P. (2019). Anti-inflammatory effects of intra-articular hyaluronic acid: A systematic review. Cartilage.

[31] Naor D., Nedvetzki S., Walmsley M., Yayon A., Turley E.A., Golan I., Caspi D., Sebban L.E., Zick Y., Garin T. (2007). CD44 involvement in autoimmune inflammations. Ann. N. Y. Acad. Sci..

[32] Lee J.H., Lee J., Seo G.H., Kim C.H., Ahn Y.S. (2007). Heparin inhibits NF-κB activation and increases cell death in cerebral endothelial cells after oxygen-glucose deprivation. J. Mol. Neurosci..

[33] Köwitsch A., Zhou G., Groth T. (2018). Medical application of glycosaminoglycans: A review. J. Tissue Eng. Regen. Med..

[34] Zhou G., Niepel M.S., Saretia S., Groth T. (2016). Reducing the inflammatory responses of biomaterials by surface modification with glycosaminoglycan multilayers. J. Biomed. Mater. Res..

[35] Kolasinska M., Krastev R., Warszynski P. (2007). Characteristics of polyelectrolyte multilayers: Effect of PEI anchoring layer and posttreatment after deposition. J. Colloid Interface Sci..

[36] Zhou G., Loppnow H., Groth T. (2015). A macrophage/fibroblast co-culture system using a cell migration chamber to study inflammatory effects of biomaterials. Acta Biomater.

[37] Bacakova L., Filova E., Parizek M., Ruml T., Svorcik V. (2011). Modulation of cell adhesion, proliferation and differentiation on materials designed for body implants. Biotechnol. Adv..

[38] Zhou G., Al-Khoury H., Groth T. (2016). Covalent immobilization of glycosaminoglycans to reduce the inflammatory effects of biomaterials. Int. J. Artif. Organs.

[39] Hernández-Montelongo J., Nascimento V.F., Murillo D., Taketa T.B., Sahoo P., de Souza A.A., Beppu M.M., Cotta M.A. (2016). Nanofilms of hyaluronan/chitosan assembled layer-by-layer: An antibacterial surface for Xylella fastidiosa. Carbohydr. Polym..

[40] Sheikh Z., Brooks P., Barzilay O., Fine N., Glogauer M. (2015). Macrophages, foreign body giant cells and their response to implantable biomaterials. Materials.

[41] Min Y.-D., Choi C.-H., Bark H., Son H.-Y., Park H.-H., Lee S., Park J.-W., Park E.-K., Shin H.-I., Kim S.-H. (2007). Quercetin inhibits expression of inflammatory cytokines through attenuation of NF-κB and p38 MAPK in HMC-1 human mast cell line. Inflamm. Res..

[42] Blackwell T.S., Blackwell T.R., Christman J.W. (1997). Impaired activation of nuclear factor-kappaB in endotoxin-tolerant rats is associated with down-regulation of chemokine gene expression and inhibition of neutrophilic lung inflammation. J. Immunol..

[43] Zhao M., Altankov G., Grabiec U., Bennett M., Salmeron-Sanchez M., Dehghani F., Groth T. (2016). Molecular composition of GAG-collagen I multilayers affects remodeling of terminal layers and osteogenic differentiation of adipose-derived stem cells. Acta Biomater..

[44] Young E. (2008). The anti-inflammatory effects of heparin and related compounds. Thromb. Res..

[45] Young E., Venner T., Ribau J., Shaughnessy S., Hirsh J., Podor T.J. (1999). The binding of unfractionated heparin and low molecular weight heparin to thrombin-activated human endothelial cells. Thromb. Res..

[46] Ruppert S.M., Hawn T.R., Arrigoni A., Wight T.N., Bollyky P.L. (2014). Tissue integrity signals communicated by high-molecular weight hyaluronan and the resolution of inflammation. Immunol. Res..

[47] Neuman M.G., Nanau R.M., Oruña L., Coto G. (2011). In vitro anti-inflammatory effects of hyaluronic acid in ethanol-induced damage in skin cells. J. Pharm. Pharm. Sci..

[48] Knudson W., Chow G., Knudson C.B. (2002). CD44-mediated uptake and degradation of hyaluronan. Matrix Biol..

[49] Yamamoto Y., Gaynor R.B. (2001). Therapeutic potential of inhibition of the NF-κB pathway in the treatment of inflammation and cancer. J. Clin. Investig..

[50] Zang Y.C., Halder J.B., Hong J., Rivera V.M., Zhang J.Z. (2002). Regulatory effects of estriol on T cell migration and cytokine profile: Inhibition of transcription factor NF-κB. J. Neuroimmunol..

[51] Köwitsch A., Abreu M.J., Chhalotre A., Hielscher M., Fischer S., Mäder K., Groth T. (2014). Synthesis of thiolated glycosaminoglycans and grafting to solid surfaces. Carbohydr. Polym..

[52] Macek M. (1993). A review cf advanced wet cleaning. Inf. Midem.

[53] Noursadeghi M., Tsang J., Haustein T., Miller R.F., Chain B.M., Katz D.R. (2008). Quantitative imaging assay for NF-κB nuclear translocation in primary human macrophages. J. Immunol. Methods.

