# Supplementary material for: Studies on the Mechanisms of Anti-Inflammatory Activity of Heparin- and Hyaluronan-Containing Multilayer Coatings—Targeting NF-κB Signalling Pathway
Source: Int J Mol Sci. 2020 May 25;21(10):3724. doi: 10.3390/ijms21103724 (PMC7279165; doi:10.3390/ijms21103724)
Supplement: Supplementary file 1 [file ijms-21-03724-s001.pdf]

Supplementary Materials

Studies on the Mechanisms of Anti-inflammatory Activity of Heparin- and Hyaluronan-containing Multilayer Coatings—Targeting NF-κB Signalling Pathway

Hala Alkhoury [1,2], Adrian Hautmann [1], Bodo Fuhrmann [2], Frank Syrowatka [2], Frank Erdmann [3], Guoying Zhou [1], Sanja Stojanović [4,5], Stevo Najman [4,5] and Thomas Groth [1,2,6,*]

[1] Department Biomedical Materials, Institute of Pharmacy, Martin Luther University Halle Wittenberg, Heinrich Damerow Strasse 4, 06120 Halle (Saale), Germany; hala.al-khoury@student.uni-halle.de (H.A.); adrian.hautmann@pharmazie.uni-halle.de (A.H.); guoying.zhou@pharmazie.uni-halle.de (G.Z.)

[2] Interdisciplinary Center of Materials Science, Martin Luther University Halle-Wittenberg, 06120 Halle (Saale), Germany; bodo.fuhrmann@cmat.uni-halle.de (B.F.); frank.syrowatka@cmat.uni-halle.de (F.S.)

[3] Pharmaceutical Biology and Pharmacology Department, Institute of Pharmacy, Martin Luther University Halle Wittenberg, Wolfgang-Langenbeck-Str. 4, 06120 Halle (Saale), Germany; frank.erdmann@pharmazie.uni-halle.de

[4] Department of Biology and Human Genetics, Faculty of Medicine, University of Niš, 18000 Niš, Serbia; s.sanja88@gmail.com (S.S.); stevo.najman@gmail.com (S.N.)

[5] Department for Cell and Tissue Engineering, Scientific Research Center for Biomedicine, Faculty of Medicine, University of Niš, 18000 Niš, Serbia

[6] Laboratory of Biomedical Nanotechnologies, Institute of Bionic Technologies and Engineering, I.M. Sechenov First Moscow State University, Trubetskaya street 8, 119991 Moscow, Russian Federation

* Correspondence: thomas.groth@pharmazie.uni-halle.de

Immunofluorescence staining (IF) of NF-kB - (Method section)

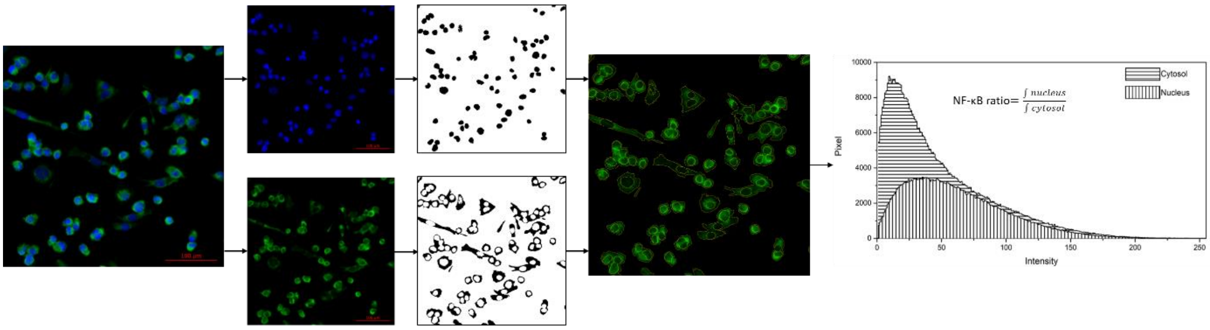

Figure S1. Nuclear and cytoplasmic regions of interests (ROIs) are applied to the original NF-κB images to extract NF-κB immunofluorescence data (a). The nuclear ROI is defined by the TO-PRO-3 mask, while the cytoplasmic ROI is illustrated by the p65 staining of NF-κB (b, blue color and green color, respectively). ImageJ software was used for the consecutive processing of the images by producing binary masks of nuclear and cytoplasmic ROIs followed by automatic thresholding to generate a binary image (c), (d). Histogram shows the frequency distribution of fluorescence intensity (e). [Scale bar: 100 μm]

Original immunoblots related to section 8

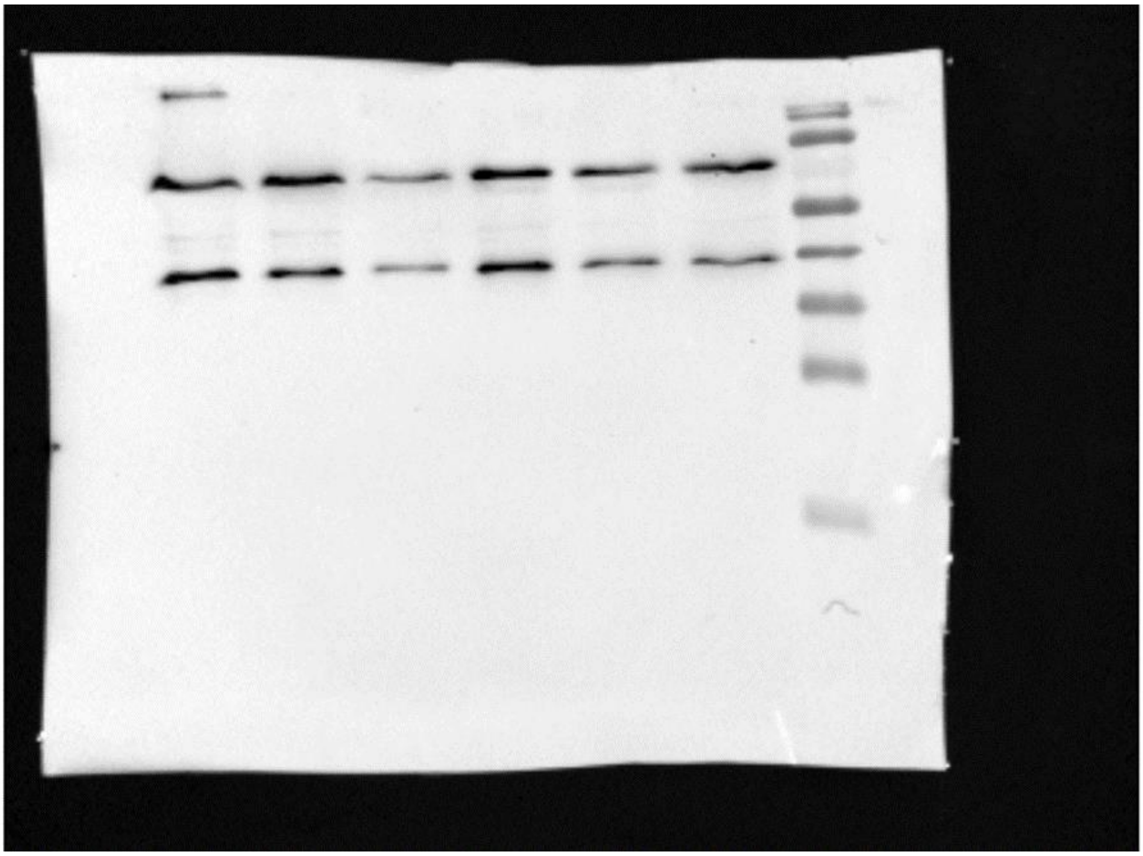

Figure S2: Original western blots' bands of p65 of NF-κB from lysates of macrophages cultured on poly (ethylene imine) (PEI) of the 1st and 2nd rows and terminal layers of PEMs

composed of hyaluronic acid (HA) or heparin (Hep) as polyanions and chitosan (Chi) as polycation abbreviated as PEI(HA/Chi)4HA of the 3rd and 4th rows and PEI(Hep/Chi)4Hep of the 5th and 6th rows. The lysates, collected after 48 h, were blotted toward (NF-κB). Duplicates of samples were measured.

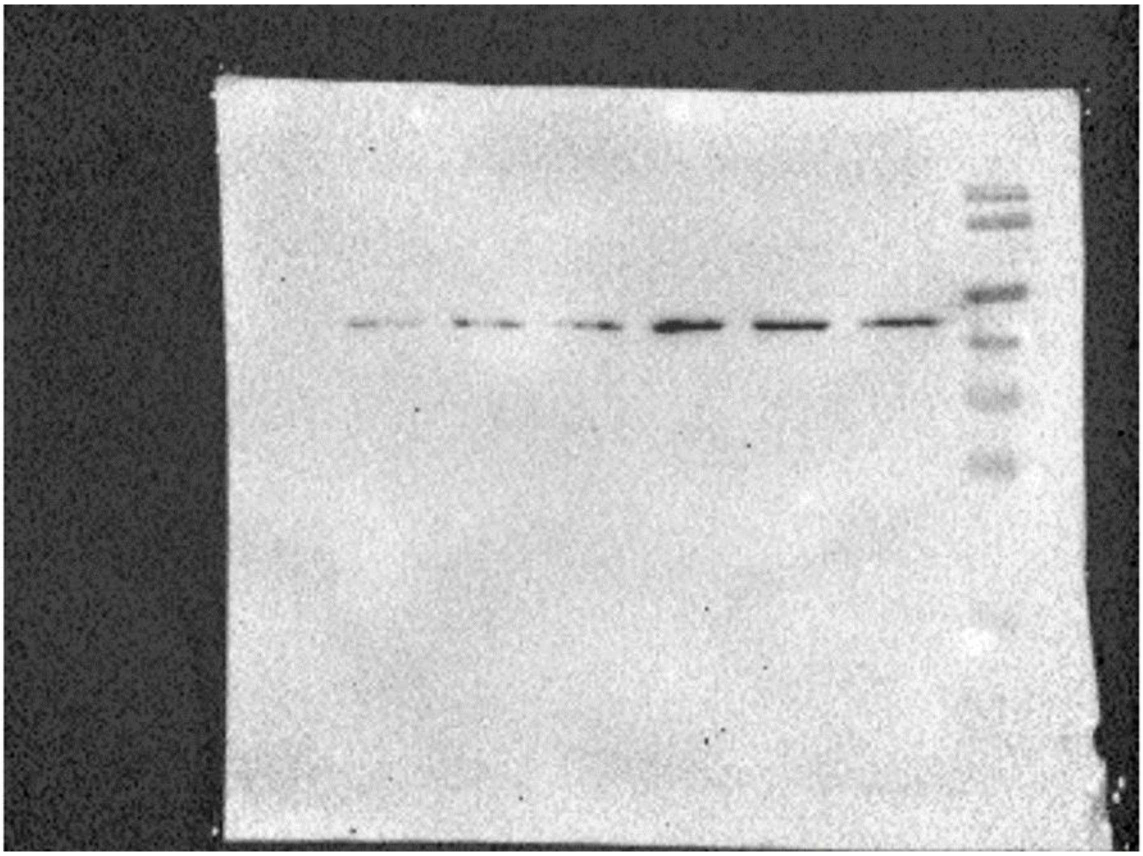

Figure S3: Original western blots' bands of actin from lysates of macrophages cultured on poly (ethylene imine) (PEI) of the 1st and 2nd rows and terminal layers of PEMs composed of hyaluronic acid (HA) or heparin (Hep) as polyanions and chitosan (Chi) as polycation abbreviated as PEI(HA/Chi)4HA of the 3rd and 4th rows and PEI(Hep/Chi)4Hep of the 5th and 6th rows. The lysates, collected after 48 h, were blotted toward actin. The immunoblotting bands were analysed by densitometry**.** Bands of p65 subunit of NF-κB in Figure S2 were normalized to expression of actin. Duplicates of samples were measured.

Survey of split images of additional cells related to section 9

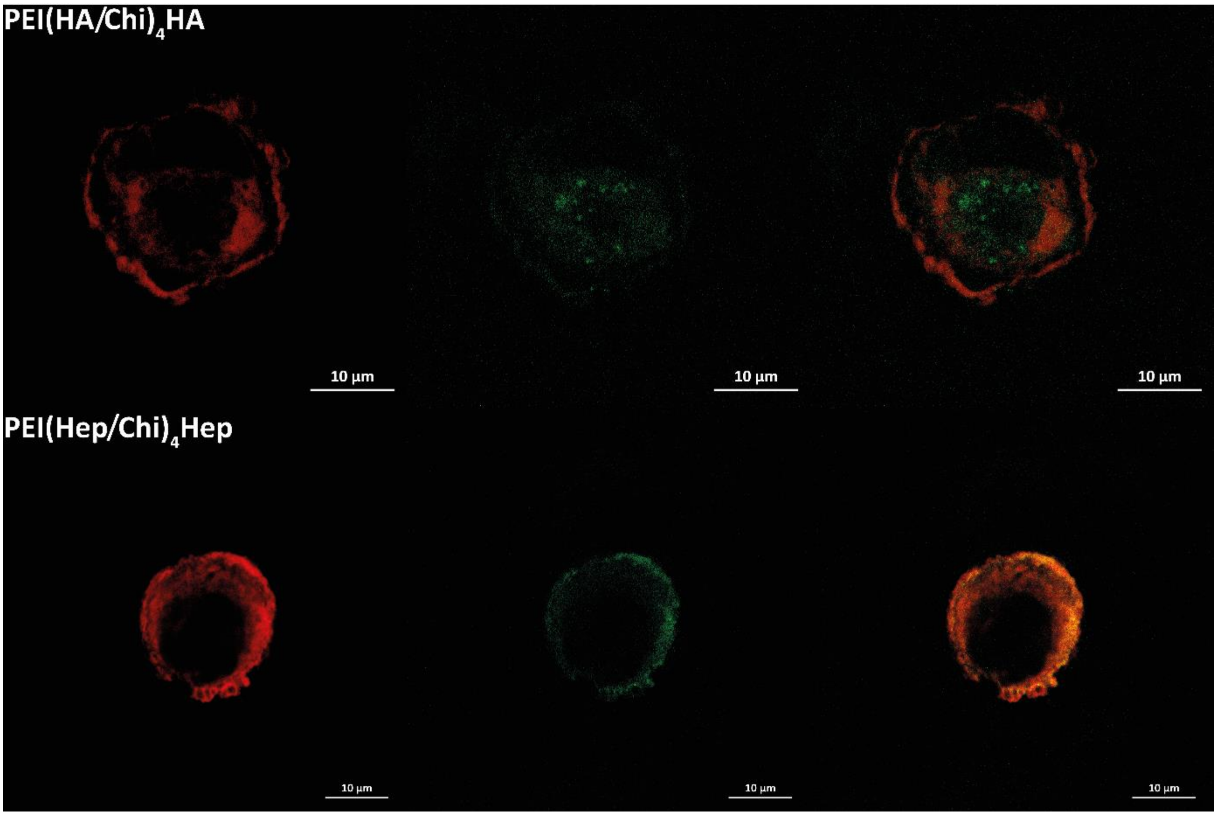

Figure S4: Representative split confocal laser microscopy (CLSM) images of adherent macrophages after 24 h incubation on terminal layers of PEM composed of hyaluronic acid (HA) or heparin (Hep) as polyanions and chitosan (Chi) as polycation abbreviated as [PEI(HA/Chi)$_4$HA, PEI(Hep/Chi)$_4$Hep], respectively. Cells were fixed with 4% paraformaldehyde and stained for DID. [63-fold oil immersion objective, Scale bar: 10 µm]

__Survey of additional 3D cell structures related to section 9__

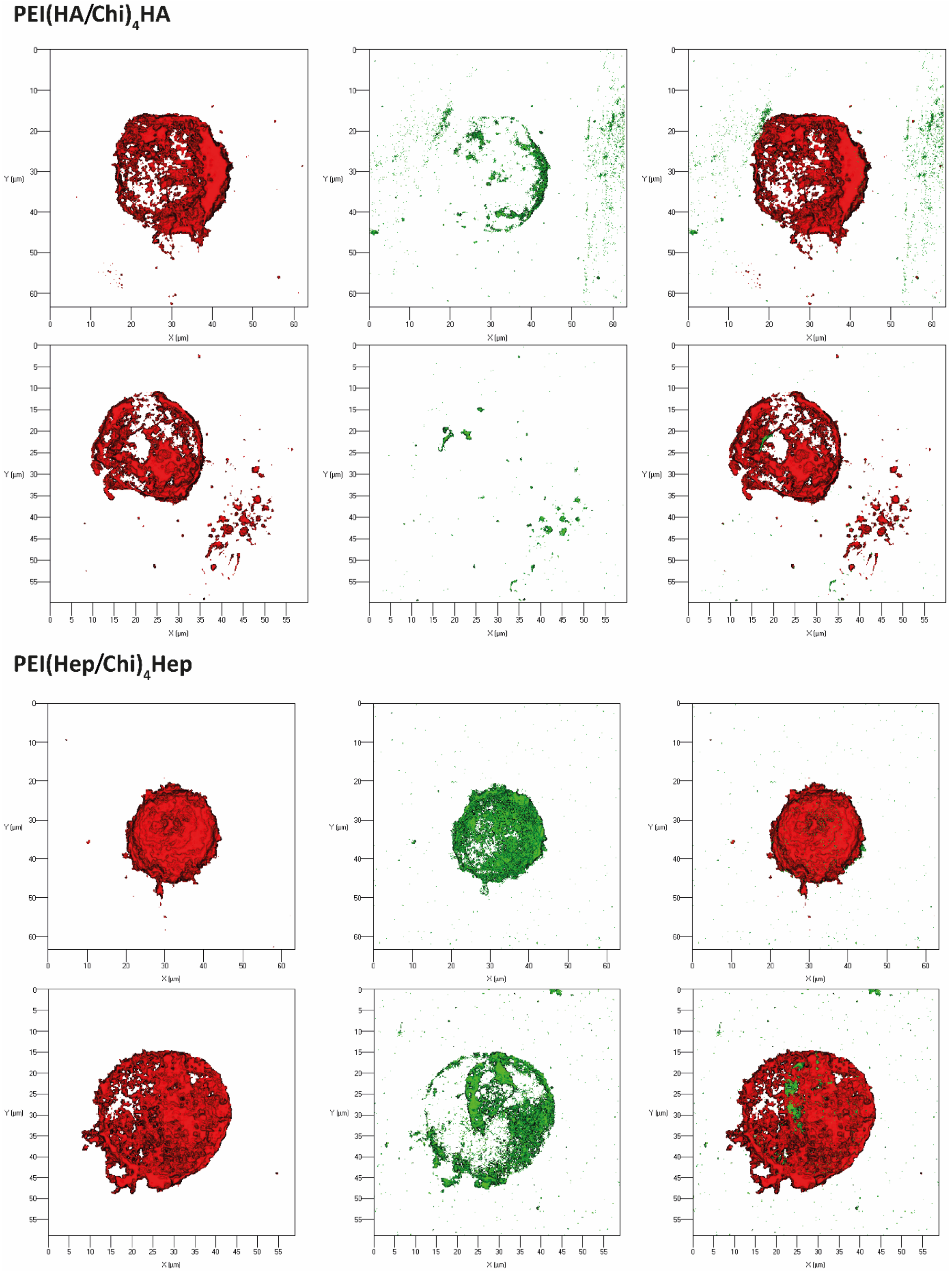

Figure S5: Representative 3D view of a z-stack in surface projection with confocal laser scanning microscopy (CLSM). Macrophages were cultured for 24 h on terminal layers of PEM composed of FITC-labelled hyaluronic acid (HA) or heparin (Hep) as polyanions and chitosan

(Chi) as polycation abbreviated as [PEI $(HA/Chi)_4HA$ and PEI $(Hep/Chi)_4Hep$], respectively. The cells were stained for DID (red, membrane staining) and the FITC-labelled GAG (green). [63-fold oil immersion objective, scale: 20 μm]. In this mode, pixel values are computed as solids which allows no transparency.
